# Dynamic Simulation of Biomechanical Behaviour of the Pelvis in the Lateral Impact Loads

**DOI:** 10.1155/2018/3083278

**Published:** 2018-09-18

**Authors:** Mohsen Hatami, Dongmei Wang, Aili Qu, Zeng Xiangsen, Qiugen Wang, Behzad Baradaran Kazemian

**Affiliations:** ^1^School of Mechanical Engineering, Shanghai Jiao Tong University, Shanghai, China; ^2^Department of Orthopedic Surgery, First People's Hospital Affiliated to Shanghai Jiao Tong University, Shanghai, China; ^3^Department of Aerospace Engineering, Sharif University of Technology, Tehran, Iran

## Abstract

The objective of this study was to develop and validate a novel 3D dynamic model of a pelvic side-impactor system. The biomechanical responses of a pelvic flexible model (having .mnf file suffix) under the lateral impact load for predicting the bone fracture mechanism are investigated as well. The 3D solid model of the side-impactor system was imported into MSC/ADAMS software for analyzing the dynamic model, and the pelvic flexible model was extracted from the CT images of a Chinese female volunteer. The flexible model of the pelvis system was developed considering a wide range of mechanical properties in the bone complex and soft tissue to achieve a realistic biomechanical response during a lateral impact. Good agreements were achieved between the dynamic simulations and the experimental results of pelvic side impacts, in terms of the biomechanical criteria. The dynamic model of impactor system could be employed to investigate the hip protector effectiveness, improving the vehicle safety, and biomechanical response of the other human organs.

## 1. Introduction

Human pelvis is an extremely stable structure and is only damaged or injured, if it is impacted by a great amount of force. It connects the upper part of the human body with the lower extremities and transfers the main load to the body. Motor vehicle crash is one of the main causes of pelvic fractures in the United States [1, 2]. Initial screening reveals that among 199,592 patients with fractures in the United States and Puerto Rico from 2002 to 2006, 61,474 patients (30.8%) had pelvic fractures [3]. Statistics shows that more than 200,000 people die in the road accidents every year in China [4, 5]. A study on the road traffic injuries (RTIs) describes that abdomen and pelvic injuries were 4.6% of the total RTIs from 2007 to 2010 in China [6]. Pelvic injury contributes significantly to traffic fatalities due to the comparatively small gap between the occupant and the intruding door. This type of casualties has long-term consequences on the society, economic aspects, and human life, indirectly [7, 8]. According to the aforementioned statistics, the investigation of the biomechanical responses of a flexible pelvis model under lateral impact load to reveal the mechanism of bone fracture is the main motivation of this study. Flexible body is an Abaqus interface for MSC-ADAMS which translates data from one or more Abaqus results (.fil) files and creates an MSC-ADAMS modal neutral (.mnf) file.

In the literature, an overall classification of these studies includes experimental tests and finite element simulations. Some researchers have studied the pelvic biomechanical response and fracture tolerances by utilizing isolated pelvises or full human cadavers and proposed different injury criteria [9–11]. These criteria include the pelvis impact force, pelvis impact energy, pelvis compression, and the impact velocity. In addition to the experimental studies, simulations using finite element (FE) models have been developed to analyze the structures and biomechanical characteristics of the pelvis under lateral impact. Plummer et al. suggested that the acetabular fracture of an isolated hemipelvis can be predicted using the finite element method [12]. Li et al. based on the FE model of a female pelvis predicted that the symphysis joint under lateral impact load exceeds normal physiological stress levels prior to the onset of bony fractures and large strains concurrently occur within the pubic ligaments [13]. Ruan et al. studied the responses of a full human body to a pelvis side impact using FE model and reported that the peak force could be used as a reasonable predictor for the pelvic injury assessment [14]. Anderson et al. reported that based on the pelvis FE model, the changes to the cortical bone thickness and elastic modulus had the largest effect on those strains [15].

Based on the aforementioned studies, some improvements still seem to be necessary. Almost all of the previous models have been developed with excessive simplifications. In fact, cortical structure varies substantially throughout the pelvis [16] and the traditional thin-shell elements may not give accurate results in some bending-dominated problems due to shear locking effects [17]. Most of the previous models include the materials such as bone, ligament, and cartilage [18], whereas muscles, skin, and fatty tissue wrapped around the bone have also a great influence on the dynamic response characteristics of the pelvis under lateral impact load. Hence, these pelvis models may need to be improved to more accurate ones and it is necessary to consider the soft tissue for the pelvis model [7, 19].

FE modelling extensively depends on the geometry and material characteristics of the objects. The model with different characteristics can generate different simulation results. Therefore, the ethnic differences in the pelvis system geometry and the material parameters have a significant effect on the biomechanical response. The parameters such as the geometrical characteristics, body mass index, weight, height, and peak bone mineral density (PBMD) are considerably different for the Chinese adult women compared to those of the American women [20]. Accordingly, these differences significantly affect the pelvis biomechanical response against the lateral impact. Since the risk of osteoporosis starts developing in a female body after the age of 46, it might be a good age criterion to select the subject of the present study [21].

Interestingly, it is estimated that rate of pelvic fractures in a woman is more than twice as high as a man [22]. North America and China have the highest rate of hip fractures in the world. It has been reported that the mean rate of hip fracture among women is 511 to 553 and 410 per 100,000 person-years in North America and China, respectively [23]. These statistics reports are the real motivations for the selection of female pelvises in this study. However, most of the studies above have focused on European or American geometry/parameters. In contrast to the previous studies, the geometric model of the pelvis is developed directly based on the CT data of a Chinese female volunteer in this research. In order to create the most accurate possible model, the surrounding soft tissue and wide range of material properties were incorporated in the bone regional variation.

In this study, an improved three-dimensional (3D) dynamic model of a pelvic side-impactor system was developed and validated in order to easily change the configuration of the simulation system according to the different models and also for reducing the simulation runtime. Moreover, this model was used to investigate the biomechanical responses of a more accurate flexible pelvis model under lateral impact load to study the bone fracture characteristics. A dynamic model of the impactor system using four different impact velocities was used to consider the impact force, compression strain, and energy as biomechanical criteria for the flexible pelvis model. The accuracy of the dynamic model of the impactor system was validated by comparing simulated results with experimental results in the literature [9].

## 2. Methodology

**Figure 1:**
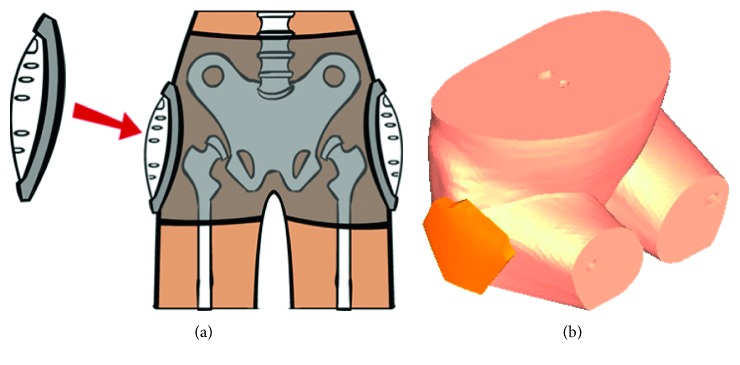
Position of the hip protector.

The 3D impactor system was firstly designed based on the spring slow compression-instant release principles, and the geometric model of a pelvis was established using CT images data. A practical simulation was designed and carried out based on the research done by Etheridge [9]. Since the force-time history curves have not been considered in Etheridge work, another simulation similar to that of Kemper [24] was also carried out in this study. The sensitivity of dynamic simulation was precisely investigated in these two cases [9, 24]. The primary component of the custom side-impactor system was equipped with a pneumatic system in cadaveric test of Kemper et al. [24], while a pair of springs was designed for the side-impactor system in this study. However, fortunately, the principle of operation (linear launch of impactor) and main factors of the experiment setup such as impact location, impactor surface, and seated position of the cadavers (two human males, 75 and 57 years old, 65 and 84 kg, 165 and 177 cm height, respectively) for both studies were approximately similar. The impactor mass and the velocity of the impactor were adjusted based on the dynamic cadaveric test of Kemper and his colleagues [24]. In addition, the first simulation was repeated using a layer of foam material as a hip protector which was fixed on the pelvis model in the position of the great trochanter as shown in Figure 1 to investigate the effect of hip protection. Parameters of the material properties of the hip protector are shown in Table 1.

### 2.1. Pelvic Side-Impactor 3D Modelling by SolidWorks

**Figure 2:**
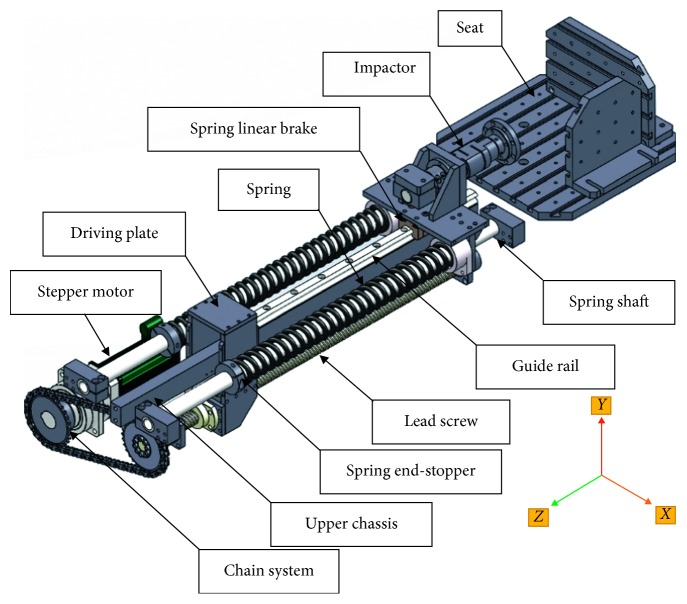
The 3D model schematic of the pelvic side-impactor system in SolidWorks.

Figure 2 shows the 3D model of the pelvic side-impactor built-in software SolidWorks® (SolidWorks Corporation, Dassault Systèmes, Vélizy-Villacoublay, Paris, France). The 3D model of the side-impactor system was designed in SolidWorks 2014 software in order to import into the software MSC-ADAMS® (Automated Dynamic Analysis of Mechanical Systems, MSC Software Corporation, version: 2013, Newport Beach, California, USA). An electromagnetic switch was fixed on the Tug-impactor of a transfer screw-nut, and an impactor was assembled on the spring linear brake.

The impactor and Tug-impactor were dragged back along a straight line by the electromagnetic switch hook. The impactor backward movement was associated with spring compression. The linear motion of the Tug-impactor was provided by transfer screw-nut, and the transfer screw-nut was driven by a chain drive. The driven sprocket was fixed at the end of the screw rod. And the drive sprocket was driven by a stepper motor. The impactor launches forward instantly, when the electromagnetic switch opened. The initial velocity of the impactor and the impact energy were determined by the length of the compression springs.

### 2.2. Dynamic Modelling of the Side-Impactor in MSC-ADAMS

The geometric model of the side-impactor system in Parasolid format was imported into the software MSC-ADAMS. The system's degrees of freedom were assigned to the joints as defined in Table 2. The model of side-impactor was simplified to prevent simulation errors and accelerate dynamic simulation (decrease computing time). The spring stiffness, material models, spring damping coefficient, joint type, and coefficient of friction were specified in MSC-ADAMS. The coefficient of stiffness and damping for both of the springs were considered 26,000 N/m and 5.0 kg/s, respectively.

The free length of the spring was 1400 mm. The coefficient of friction between the seat and the guide rail was considered equal to 0.05 [9]. The mass of the impactor was 22.1 kg [9]. The radius and height of the impactor were 50 mm and 100 mm, respectively. The impactor linear motion was given in *Z* direction. In order to coordinate the actuator components and stepper motor, a step function was applied to the dynamic simulation of the pelvic side-impactor system.

### 2.3. Flexible Body Model of the Pelvis

A serial of computed tomography (CT) scans with an XY solution of 512 × 512 was obtained from a Chinese female volunteer (49 years old, 60 kg, 166 cm) because the biomechanical responses can be more precise depending on the accuracy of the pelvis model. The pelvic geometry model was constructed from the L4 vertebral body to the middle of the femur (the project was approved by ethical committees of the Shanghai 1st People Hospital, and the volunteer gave informed consent to the work). The images were imported into software Mimics®(Materialise's interactive medical image control system, version: 10.1, Leuven, Belgium) to get the triangular format of pelvic complex surface. Therefore, the structure of bone, L4 vertebral body, skin, and pelvic arteries were extracted from the Mimics 10.1 software and exported in STL format model. And then, the STL model was imported into Geomagic® Studio software (version: 10, North Carolina, USA) in order to establish the 3D surface model.

Finally, the surface model of the pelvic-femur-soft tissue complex was imported into software Hypermesh® (version11, USA) to mesh and define the mechanical properties of tissue materials.  Figure 4 shows the geometrical model of pelvic-femur-soft tissue complex and the position of the soft tissue thickness over the greater trochanter. CT images were scanned with the patient in supine posture, and some changes were made so as to simulate the seated posture. In order to obtain the pelvis model under sitting posture, the skin model was cut near the groin and the femur of the model was rotated 90° around the lateral-medial axis of the femur with the rotation center of the femoral head.

The thickness parameters of the soft tissue around the ischial tuberosity were considered [26]. And the thickness of the soft tissue overlapping the greater trochanter was defined based on previously published data [9, 27]. The surfaces were then tangentially connected between the back and buttocks. The cartilage models in hip joint and sacroiliac joint were created according to the gap between two joint surfaces [28]. The rotated parts of the pelvic skin were stitched together after the femur rotation. The intervertebral disc model was also created between the fifth lumbar vertebra and sacrum. The thickness of the pelvic artery wall was set equal to 0.8 mm [29]. Trochanteric soft tissue thickness (*T*) was determined 3.28 cm. The bone, artery, intervertebral disc, cartilage, and peripheral soft tissue were meshed by tetrahedron elements, and the sacroiliac ligament, sacral ligament, sacral spine ligament, pubic ligament, and iliofemoral ligament were created using link elements according to their anatomical attachment points on the bone.

### 2.4. Model Mesh

For estimating the optimal mesh size, a tetrahedron grid which is proper to model the complex geometries was used here to mesh the pelvis contours. Bony and cartilage tissue, artery, and soft tissues had the optimal average mesh sizes of 3 mm, 1.5 mm, and 8 mm, respectively. The total quantity of the elements and nodes which are used to model the geometry is 246954 and 60950, respectively. The detailed list of the quantity of the elements and nodes and also the type of the elements used in each part of the model are demonstrated in Table 3.

### 2.5. Material and Properties

We modeled the bone as a heterogeneous fragmented linear plastic material whose characteristics have been specified element by element using the method proposed by Morgan et al. [30]. A particular relation between the stress and the strain has also been employed to model the massive compression of the bone [31]. The characteristics of the material are listed in Table 4–6.

As it can be seen in Table 4, the material properties of the bone tissue were assigned according to the CT Hounsfield unit of CT images, the mathematical relation between Young's modulus, and apparent density [32–34,42].

The material parameters for all the sections of the pelvis are presented in Table 5. Due to the complexity of pelvic system, the ligaments were simplified with rod elements. Hyperelastic material properties were assigned to the artery, cartilage, and the other soft tissues of the pelvic system as reported in the literature [43]. The properties of the ligaments are presented in Table 6. The pelvis mass was reached 12 kg after designating the parameter values for all sections of the pelvis.

The meshed model of the pelvic-femur-soft tissue complex in .inp format (the input data file format in Abaqus) was imported into software Abaqus® (Dassault Systèmes Simulia Corp, Dassault Systèmes, Johnston, Rhode Island, United States) in order to create a .mnf format model, which can be imported into MSC/ADAMS software and converted to a flexible body model of the pelvic-femur-soft tissue complex.

### 2.6. Boundary Condition

**Figure 3:**
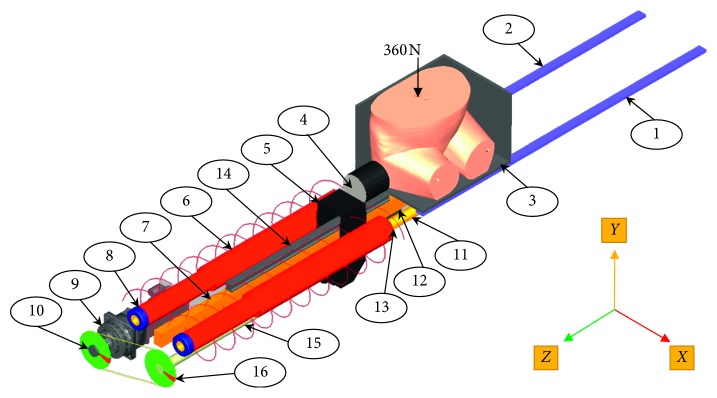
Configuration of the pelvic side-impactor dynamic system in MSC-ADAMS.

**Figure 4:**
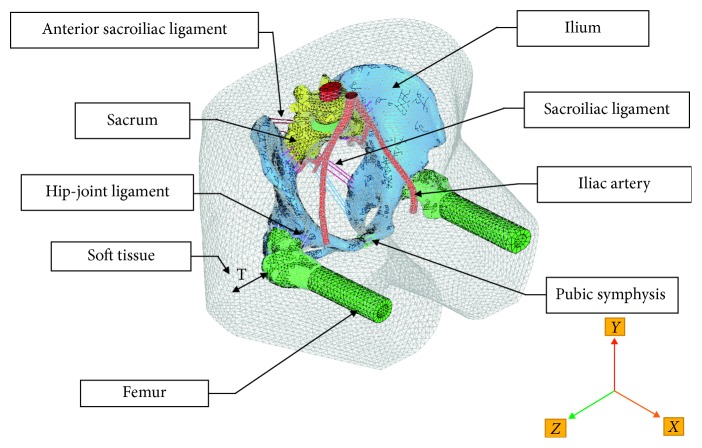
The meshed model of pelvic-femur-soft tissue complex with the internal components.

The following boundary conditions are used in this simulation. The impacts velocities are imposed as shown in Table 7. A static preload of 360 N is imposed using a fixed joint on the top of the pelvis model to simulate the weight of the upper part of the human body which is equal to 60% of the whole body weight as depicted in Figure 3 [9]. This external loading is fixed on the vertebral body exactly on the top of the lumbar vertebra (L5). The friction coefficient between the pelvis and the seat is equal to 0.294. The flexible body model was set up on the seat of the impactor system, aligning the greater trochanter with the center of the impactor. The contact relation was defined between the pelvic model and the seat model.

### 2.7. Impact Simulation and Validation

In order to validate the dynamic simulation of the pelvic side-impactor, the velocity of the impactor was adjusted very close to experiment [9]. Therefore, the velocities of the impactor were considered to be 2.5, 3.0, 3.3, and 5 m/s. The maximum pelvic compression was denoted by *C*_max_ which can be calculated as the following equation [9]:

(1) $$\begin{array}{c} C_{\max}=\frac{\Delta L_{\max}}{L_{0}}\times 100\%, \end{array}$$

where *L*_0_ is the initial distance between the left and right sides of the greater trochanter. Δ*L*_max_ is the change of *L*_0_ during the impact. *F*_max_ and *E*_peak_ are defined as the maximum force and maximum energy recorded during the impact, respectively. The impact simulations were performed in MSC-ADAMS. The total runtime of the dynamic simulation and calculation was 15 minutes. According to the results obtained from the simulation, the changes of *C*_max_, *F*_max_, and *E*_peak_ were obtained with respect to the impact velocities. The biomechanical response characteristics of the pelvic-femur-soft tissue complex were discussed based on the analyzed results. In addition, a comparative analysis was carried out in order to validate the dynamic model of the pelvic side-impactor system and pelvic complex model.

## 3. Results

### 3.1. Impact Force

**Figure 5:**
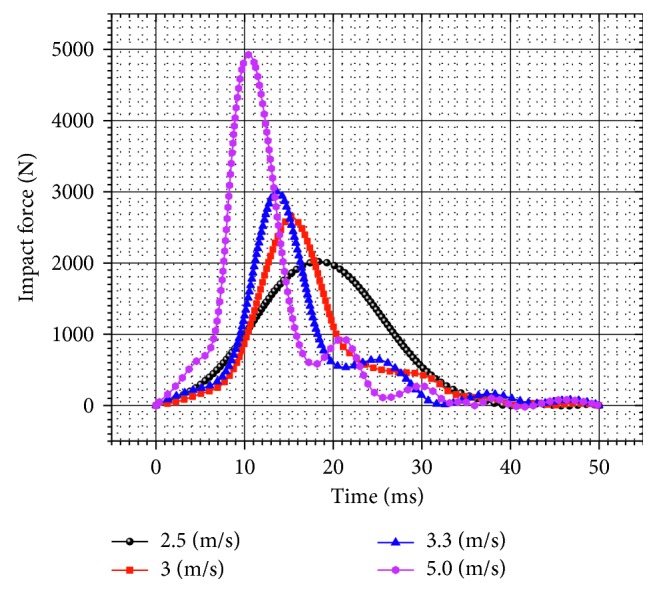
Force-time curves for the unpadded hip impact.

The impact force for the unpadded hip impact is shown in Figure 5. The peak value of the impact force becomes larger and appears earlier with the increase of the impact velocity. However, the duration of the impact process decreases. The *F*_max_ reaches to 2018.7 N within 18.3 ms, when the impact velocity is 2.5 m/s. On the other hand, *F*_max_ reaches to 3005.8 N within 13.6 ms, when the impact velocity is 3.3 m/s. According to the fracture criterion (bone yield strength), the bone fracture occurs at the impact velocity of 5 m/s. The value of the impact force reaches to 4924.9 N upon the impact velocity of 5.0 m/s and the bone fracture occurs within 10.4 ms.

The responses of the impact forces applied to the pelvic flexible model were compared with both the nonfracture and fracture response ranges of the pelvis impact forces used in Etheridge's and his colleagues' experimentation [9]. Although this experiment has not been carried out for the velocity of 2.5 m/s in [9], since there are many other cases such as pelvic injuries due to falling [25], in which this approximate impact velocity is important therefore, this impact velocity has also been investigated in this study.

### 3.2. Compression Ratio

**Figure 6:**
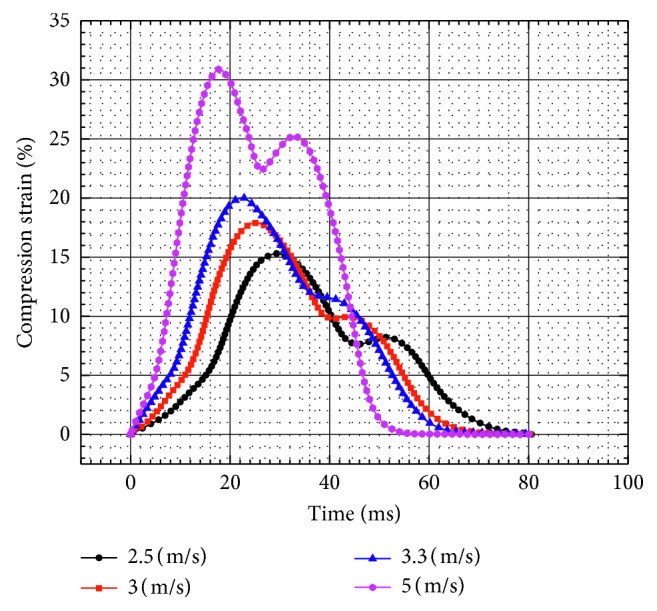
Compression ratio-time curves for the unpadded hip impact.

**Figure 7:**
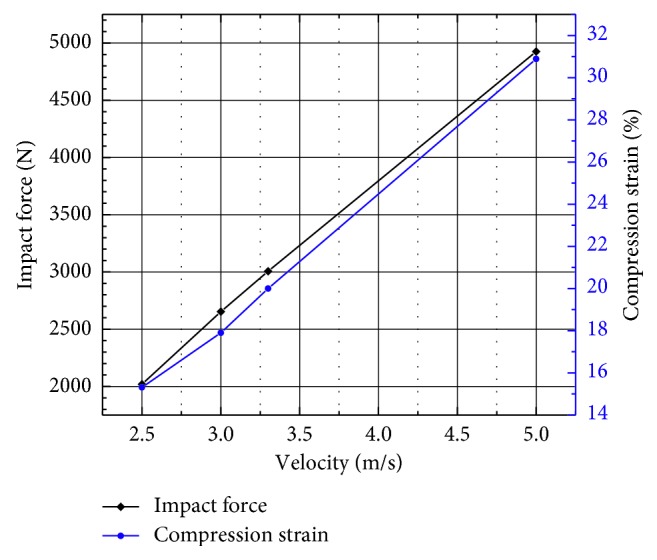
Compression ratio-impact force in different velocities for the unpadded hip impact.

The transverse compression ratio of the pelvis is shown in Figure 6. It was observed that the maximum compression ratio exponentially increases with the increase in the impact velocity as shown in Figure 7. When the impact velocity is 5 m/s, *C*_max_ increases to 30.88% and its corresponding time decreases to 17.67 ms with the increase in the impact velocity.

### 3.3. Model Validation

As shown in Table 7, the results of the dynamic simulation were compared with those of the dynamic test of Etheridge and his colleagues [9]. The findings were indicated that *C*_max_ and *E*_peak_ were close to the average and also within the lower and upper limits of the experimental results. However, the maximum impact force of the pelvic flexible model was higher than the other various tests results. The oscillation of the impact force data and individual differences in the pelvis model such as specimen age, bone density, and pelvic morphology were observed as the main reasons for the difference in the maximum impact force. As a specific example in Table 7, the energy dissipated at peak force for the velocity of 3 m/s deviates from the normal trend. This significant difference can be ascribed to the structural and anthropometric differences between the different cadavers which is inevitable in the experimental studies.

**Figure 8:**
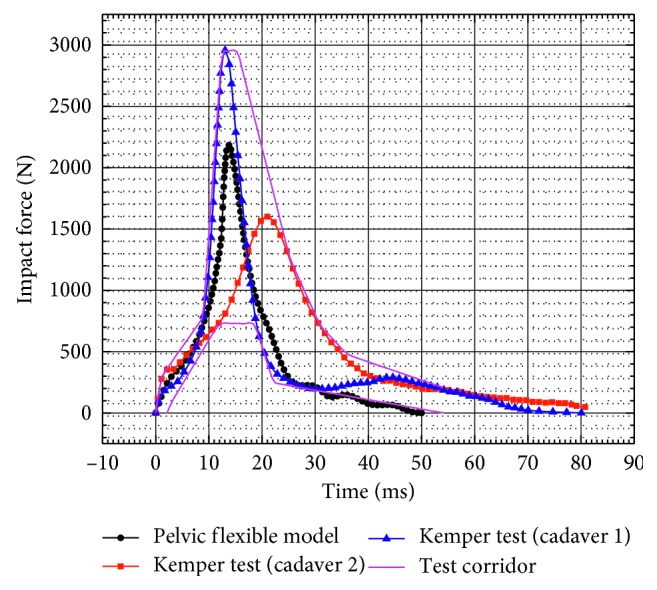
Force-time curve for the unpadded hip impact.

In order to further improve the accuracy of the pelvis side-impactor dynamic simulation and due to the absence of force-time history curves in the study of Etheridge, the dynamic simulation was carried out based on the Kemper test. The impactor mass was changed to 16 kg, and the velocity of the impactor was considered equal to 3 m/s based on the dynamic test of Kemper and his colleagues [11].  Figure 8 shows that the force-time curve predicted by the pelvic flexible model lies within the test corridor developed by Kemper et al. which indicates the validity of this dynamic simulation [11]. In the tests, the range of peak force and the corresponding time were 2278.86 ± 6.77 N and 17.02 ± 3.97 ms, respectively. In the dynamic simulation, the peak force of the pelvic flexible model is 2183.7 N within a time of 13.8 ms. The results show that the peak force of pelvic flexible model is within the range of the tests.

### 3.4. Sensitivity to Load Conditions

Based on the dynamic test performed by Etheridge and his colleagues [9], another 3D dynamic numerical model under the impact velocities of 2.5, 3, 3.3, and 5 m/s was developed by utilizing the pelvic flexible model.  Table 8 lists the peak force, maximum compression, maximum energy, and injuries of pelvis in these simulations [9]. As the impactor velocity increases, the peak impact force and maximum compression increase accordingly. In addition, the injuries of the pelvis become more and more serious. Specifically, at the velocity of 5.0 m/s, a fatally displaced fracture happened in the pubic rami. These findings indicate that the changes in impactor velocity had a significant effect on the pelvic injury in side impacts.

**Figure 9:**
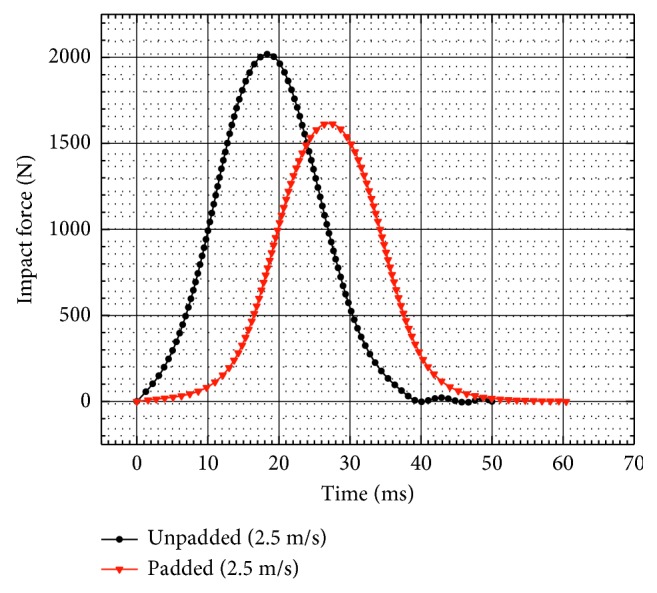
Influence of hip padding on the impact force of the pelvis.

Figure 9 shows the comparison of impact force under padded and unpadded loads. Obviously, the reaction force curve under padded load has found a little decrease in amplitude and an increase in period. In addition, the peak force in padded load simulation was smaller. In fact, the padding attached to the pelvis played a significant role in absorbing the impact energy and alleviating the injuries of the pelvis.

## 4. Discussion

Side impact dynamic simulation is necessary to make the vehicle safe from this prevalent cause of injury. Side impacts can produce head, neck, pelvis, and thorax injuries due to sudden acceleration and interior contact with a collapsed interior. However, for the frontal impacts, there are some restraints such as seat belt, and frontal airbags which can effectively protect the occupant from these injury pathways. This study is useful to design and create a dynamic model of the side-impactor and pelvic complex system that can be applied to simulate the biomechanical response performance of the pelvic complex under lateral impact with least possible time, acceptable accuracy, and high flexibility.

### 4.1. Model Creating

A pelvic impactor using the spring compression release system was designed in this study. The dynamic model of the impactor was created in MSC/ADAMS software. The spring compression, energy storage, and instant release were simulated which provided the initial velocity and the impact energy. The simulation results had a great significance for optimizing the design and motion control software for the development of impact test device. It is known that the body mass index, weight, and height of the Chinese woman is 22.6 ± 3.2 kg/m^2^, 54.0 ± 8.9 kg, and 154.5 ± 6.1 cm, respectively, whereas that of the Caucasian American woman is 27.0 ± 6.4 kg/m^2^, 71.7 ± 17.3 kg, and 163.0 ± 6.2 cm, respectively [20]. In addition, it has been demonstrated that the PBMD for Chinese women at the lumbar spine and various sites of the hip is 5.1% ± 2.7% (whose range is 0.5–7.2%) lower than those for Caucasian American women. However, the age of osteoporosis occurrence at the total hip, intertrochanter, and trochanter in both of races is about 46 [21]. Due to this reason, pelvic geometry model is constructed on the basis of CT image of a 49-year-old female. Some physical factors, such as the rotational inertia, turning radius, and the position of the center of mass, can be affected by these dissimilarities. These differences have also noticeable influences on the biomechanical response of the pelvis under lateral impact loading. Therefore, geometric model of the pelvis is directly developed based on the CT data of the Chinese female volunteer.

The material properties of the bone were set according to Hounsfield unit of the bone CT images. The dynamic model of the pelvic flexible model had higher geometric, anatomical, and material similarity than the previous work [18]. The coefficient of friction between the impactor and the pelvis surface around the greater trochanter, and between pelvic model surface around ischium and the seat was considered equal to 0.294. This coefficient is also the same for those of the contralateral side of the impactor. This coefficient can also be considered for the lower sliding rails of the seat [44].

### 4.2. Contact Force and Compression Ratio

In this research, the energy or initial velocity of the impact puncher depended on the compression of the springs. In order to validate the dynamic model, the simulation conditions were consistently based on the experimental conditions of our intended reference [9]. The pelvic flexible models' accuracies in predicting pelvic injury were compared with the dynamic test. With the most accurate model (flexible model of the pelvis), sensitivity studies were carried out to analyze the biomechanical response and injury of the pelvis. The simulation and experimental results of *F*_max_, *C*_max_, and *E*_peak_ were in good agreement and indicate that the dynamic model of the pelvic side-impactor and pelvic flexible model was precise and reliable. The contact area on which the load was applied in the present study (78.5  cm^2^) concentrated exactly over the greater trochanter of the femur. The time corresponding to peak load in the present study was approximately 50 ms as shown in Figure 5, which was consistent with the time reported in the literature [10, 11, 45].

The sensitivity study of the loading conditions indicated that pelvic injury under unpadded load was more serious than that of under padded load. This revealed that the development and installation of some appropriate energy absorbers or padding between the car door and the occupant can significantly reduce pelvic injury and will be beneficial for occupant protection. From the sensitivity study of both the impactor velocity and the pelvis with/without padding, it can be observed that the pelvis impacted under a higher peak force will sustain more serious injury. This phenomenon can also be seen in the dynamic test simulations of the pelvis flexible model. Therefore, we can infer that the peak impact force can be used as a reasonable predictor for pelvic injury assessment, which agrees with the study of Etheridge et al. [9].

### 4.3. Applications and Limitations

The dynamic model created in MSC/ADAMS has the feasibility of quick change of the parameters such as materials of machine components while having lower simulation time and reasonable accuracy in the dynamic analysis. The computing time for impact simulation using the finite element model of the pelvis in the literature is approximately 17 hours, while the computing time for the same impact simulation in MSC/ADAMS is no more than 15 minutes, using a 16-CPU processor [18, 36, 45].

**Figure 10:**
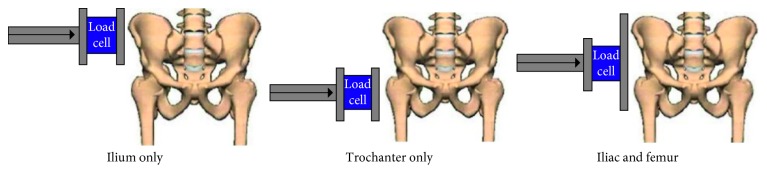
The new simulation task through changing location of the impactor.

Although the cadaver test is an ideal method to study the dynamic response of the pelvis, the cadaver pelvis is difficult to collect and preserve while the cadaver specimen number is limited and it cannot be reused. Since there will be errors in experimental data acquisition, the dynamic simulation of the pelvic side-impactor can be effective. In addition, a new simulation task can easily be performed by changing the position of the impactor relative to the pelvic complex as shown in Figure 10 [11].

The biomechanical responses of the pelvic complex with different degrees of osteoporosis under impacting load can also be simulated and investigated using the dynamic model. More importantly, the model of a special protector can easily be added in the dynamic model and the biomechanical effects of the protector under impact load can be analyzed by dynamic simulation. The simulation results are helpful for optimal design of a hip protector. This impact test setup can be used not only for biomechanical applications and materials, but also for the industrial impact analyses such as external impact of various pipes and rigid plastic sheets. Despite the achievements above, there are yet some limitations in this study. First are the differences between the pelvis model and the test specimens. In addition to the differences in dimensions, the differences in material characteristics are pretty significant. In this study, the pelvis model is created according to the CT data of a volunteer Chinese female. However, the selected tests are performed by utilizing the pelvic specimens of an American person.

## 5. Conclusion

Increasing the accuracy and reliability of the dynamic model of the impactor system and the pelvic complex model was considered and successfully validated in this study. And the present results suggest the effect of the impact force and compression on pelvic response for female model in automotive side impacts. In addition to the pelvic complex model, the dynamic model of the impactor system can be used to investigate the biomechanical response from other organs of the human body such as thorax and lower leg under lateral impact. Moreover, the simulation results can be helpful to design the protector to prevent the pelvic fracture. The dynamic model can also be employed to evaluate the biomechanical effects of the protector during lateral impact using dynamic simulation calculation and analysis.

## Figures and Tables

**Table 1:** Material properties of the hip protector.

Material/parameters	Thickness (cm)	Density (g/cm^3^)	Elastic modulus (MPa)	Poisson ratio
Hip protector (foam)	1.8	1.8	10 [25]	0.3

**Table 2:** Topology model for pelvic side-impactor system.

Component number	Component name	Connected part number	Joint	Degree of freedom	Joint image
1 and 2	Linear guide rails	Ground	Fixed joint	0	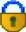
3	Seat	1 and 2	Translational joint	1 translational	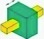
4	Impactor	14	Translational joint	1 translational	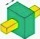
5	Driving plate	12 and 15	Screw joint	1 translational	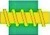 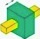
6	Spring	8 and 13	Translational joint	1 translational	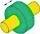
7	Upper chassis	Ground	Fixed joint	0	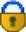
8	Spring end-stopper	11	Fixed joint	0	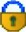
9	Electromotor	Ground	Fixed joint	0	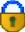
10	Motor drive	16	Revolute joint	1 rotational	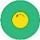
11	Spring shaft	Ground	Fixed joint	0	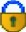
12	Lower guide rail	7	Fixed joint	0	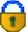
13	Spring keeper	11	Translational joint	1 translational	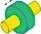
14	Upper guide rail	7	Fixed joint	0	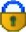
15	Lead screw	5	Screw joint	1 translational	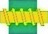
16	Chain system	15	Revolute joint	1 rotational	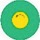

**Table 3:** Number of elements and nodes of the pelvis model.

Section	Elements	Nodes	Element type
Sacrum	25396	6396	Tetrahedron
Ilium	41351	10855	Tetrahedron
Femur	15185	5087	Tetrahedron
Soft tissue	165022	38612	Tetrahedron

**Table 4:** Material properties of solid bone elements.

Pelvic parts	Elastic modulus *E*=*Aρ*^*B*^ (MPa)				Yield strength *σ*_*y*_=*Cρ*^*D*^ (MPa)			
	*A*	*B*	min	max	*C*	*D*	min	max
Ilium [12, 32]	2890	2.63	3.6	12214	32.4	1.85	2	115
Sacrum and lumbar vertebra [33, 34]	4730	1.56	3	11900	37.1	1.74	1	104
Femur [33, 34]	6850	1.49	159	18500	85.5	2.26	2	505
Bone expression [35]	*ρ*=0.00115HU − 0.00242							

*Note*. Poisson's ratio of the bone tissue is 0.3 and postyield modulus *E*_*t*_ is considered equal to 10% of the elastic modulus *E* [42]. There is a linear relation between bone density (unit=g/cm^3^) and CT (unit=HU) value, and this formula has been obtained by [35].

**Table 5:** Material properties of the soft tissue elements.

Pelvis parts	Constitutive model	Material parameters	Density (g/cm^3^)
Pubic symphysis [36]	Three-parameter Mooney–Rivlin hyperelastic	*C*_10_=0.1 MPa *C*_01_=0.45 MPa *C*_11_=0.6 MPa	1.2
Sacroiliac cartilage [36]	Two-parameter Mooney–Rivlin hyperelastic	*C*_1_=4.1 MPa *C*_2_=0.41 MPa	1.2
Hip joint cartilage [36]	Two-parameter Mooney–Rivlin hyperelastic	*C*_1_=4.1 MPa *C*_2_=0.41 MPa	1.2
Annulus (fiber) [37]	Linear elastic	*Ε*=450 MPa υ=0.45	1.1
Nucleus [38]	Linear elastic	*Ε*=2.25 MPa	—
υ=0.49	1.1	—	—
Artery [39]	Three-parameter Mooney–Rivlin hyperelastic	*C*_10_=18.9 kpa *C*_01_=2.75 kpa *C*_11_=857 kPa	1
Surrounding soft tissue [35]	Two-parameter Mooney–Rivlin hyperelastic	*C*_10_=85.5 kpa *C*_01_=21.38 kpa	0.749

**Table 6:** Connector elements of ligaments with elastic behaviour.

Position of pelvic ligaments	Number of elements	Stiffness (N/m) [28]
Anterior sacroiliac ligament	14	700
Short posterior sacroiliac ligament	8	400
Long posterior sacroiliac ligament	6	1000
Interosseous sacroiliac ligament	22	2800
Sacroiliac ligament nodes	6	1500
Sacrospinous ligament [28]	6	1400
Pubic ligament [40]	8	543
Iliofemoral ligament [41]	30	3000

**Table 7:** Values of *F*_max_, *C*_max_, and *E*_peak_ in the different velocities.

	*V*_*t*_ (m/s)		*E*_peak_ (J)		*F*_max_ (N)		*C*_max_ (%)	
	Simulation	Experimental	Simulation	Experimental	Simulation	Experimental	Simulation	Experimental
	2.50	—	32.13	—	2018.70	—	15.31	—
	3.00	3.19	40.12	74.50	2653.00	1600.00	17.90	17.66
	3.30	3.30	43.93	45.52	3005.80	2003.00	20.00	20.82
	5.00	5.00	86.62	82.32	4924.90	3360.00	30.88	31.88
Mean ± SD	3.45 ± 1.08	3.83 ± 1.01	17.96 ± 24.45	67.45 ± 19.39	3150.60 ± 1251.39	2321 ± 922.09	21.02 ± 6.84	23.45 ± 7.47

**Table 8:** Impact response and injuries of the pelvis under different impact velocities.

Impactor velocity *V*_*t*_ (m/s)	Max energy *E*_peak_ (J)	Peak force *F*_max_ (N)	Max compression *C*_max_ (%)	Fractures
3.3	45.52	2003	20.82	None
5	82.32	3360	31.88	L. sacral ala, post-iliac crest, sacral ala, and inf/sup rami.
				R. inf/sup rami, inf rami, and pubis.

L: left side; R: right side; inf/sup: inferior and superior.

## Data Availability

The data used to support the findings of this study are available from the corresponding author upon request.
